# Kidney transplantation in a patient with HIV disease

**DOI:** 10.4103/0971-4065.57113

**Published:** 2009-07

**Authors:** L. I. Gonzalez-Granado

**Affiliations:** Immunodeficiencies Unit, Hospital 12 de Octubre, Avenida Andalucia S/N, 28041, Madrid, Spain

Sir,

I have read with great interest the article from Bansai *et al*.[1] I agree that we are now able to offer good alternatives to HIV patients transplanting kidney or liver apart from conservative management. However, I disagree with the antiretroviral regimen. It is well known that new PI's are preferable than old ones and nevirapine.[2] The only point is that you need to check serum antiretroviral levels at least for several weeks after kidney transplantation in order to achieve a suitable dose and dose interval. For instance, with lopinavir/ritonavir (LPV/r), frequently a more prolonged interval of dose and dose lowering is needed. Today, nevirapine is used only in children below 6 months of age, women with CD4 count below 250 cells/mm^3^ and men with less than 400 cells/mm^3^. With higher CD4 count, severe hepatotoxicity has been described. In some cases, hepatic injuries continued to progress despite discontinuation of nevirapine.[3] International guidelines do not recommend the regimen for this patient.[4,5]

I would like to emphasize that serum levels of antiretroviral drugs may help to achieve the best outcome for kidney transplantation in HIV patients.
